# European Glaucoma Society research priorities for glaucoma care

**DOI:** 10.1136/bjo-2023-323648

**Published:** 2023-11-03

**Authors:** Augusto Azuara-Blanco, Noleen McCorry, Andrew J Tatham, Stelios Georgoulas, Panayiota Founti, Cedric Schweitzer, Frances Meier-Gibbons, Philippe Denis, Anja Tuulonen, Gauti Johannesson, José María Martínez de la Casa, Verena Prokosch, Dimitrios A Giannoulis, Luis Abegão Pinto, David Garway-Heath, Fotis Topouzis

**Affiliations:** 1 Centre for Public Health, Queen's University Belfast, Belfast, UK; 2 Ophthalmology Department, Princess Alexandra Eye Pavilion, Edinburgh, UK; 3 Ophthalmology, Cambridge University Hospitals NHS Foundation Trust, Cambridge, UK; 4 Glaucoma Service, Moorfields Eye Hospital City Road Campus, London, UK; 5 Ophthalmology, University Hospital Centre Bordeaux, Bordeaux, France; 6 Ophthalmology, Eye Center Rapperswil, Rapperswi, Switzerland; 7 Service d'Ophtalmologie, Hôpital Universitaire de la Croix-Rousse, Hospices Civils de Lyon, Lyon, France; 8 Tays Eye Centre, Tampere University Hospital, Tampere, Finland; 9 Department of Clinical Sciences, Ophthalmology, Umeå University, Umea, Sweden; 10 Ophthalmology, Hospital Clinico San Carlos, Madrid, Spain; 11 Department of Ophthalmology, University of Cologne, Koln, Germany; 12 First Department of Ophthalmology, School of Medicine, Faculty of Health Sciences, Aristotle University of Thessaloniki, Thessaloniki, Greece; 13 Department of Ophthalmology, Santa Maria Hospital, University of Lisbon, Lisboa, Portugal; 14 Glaucoma Service, Moorfields Eye Hospital NHS Foundation Trust, London, UK

**Keywords:** glaucoma, clinical trial, diagnostic tests/investigation, treatment medical, treatment surgery

## Abstract

**Background/Aims:**

The goal of health research is to improve patients care and outcomes. Thus, it is essential that research addresses questions that are important to patients and clinicians. The aim of this study was to develop a list of priorities for glaucoma research involving stakeholders from different countries in Europe.

**Methods:**

We used a three-phase method, including a two-round electronic Delphi survey and a workshop. The clinician and patient electronic surveys were conducted in parallel and independently. For phase I, the survey was distributed to patients from 27 European countries in 6 different languages, and to European Glaucoma Society members, ophthalmologists with expertise in glaucoma care, asking to name up to five research priorities. During phase II, participants were asked to rank the questions identified in phase I using a Likert scale. Phase III was a 1 day workshop with patients and clinicians. The purpose was to make decisions about the 10 most important research priorities using the top 20 priorities identified by patients and clinicians.

**Results:**

In phase I, 308 patients and 150 clinicians were involved. In phase II, the highest-ranking priority for both patients and clinicians was ‘treatments to restore vision’. In phase III, eight patients and four clinicians were involved. The top three priorities were ‘treatments to stop sight loss’, ‘treatments to restore vision’ and ‘improved detection of worsening glaucoma’.

**Conclusion:**

We have developed a list of priorities for glaucoma research involving clinicians and patients from different European countries that will help guide research efforts and investment.

**WHAT IS ALREADY KNOWN ON THIS TOPIC**

- Identification of research priorities needs to involve relevant stakeholders.

**WHAT THIS STUDY ADDS**

- The top 10 research priorities for glaucoma have been identified.

**HOW THIS STUDY MIGHT AFFECT RESEARCH, PRACTICE OR POLICY**

- This study will influence future research strategies and funding opportunities.

## Introduction

Glaucoma is among the leading causes of vision impairment in Europe and, in the recent past, we have seen the incorporation of technologies that aim to improve glaucoma care.^1^ However, there are many questions regarding glaucoma management (eg, diagnosis, evaluation of risk, treatment, models of eye care) that remain unanswered.

The ultimate goal of health research is to improve patient care and outcomes. Thus, it is essential that research addresses questions that are important to patients and clinicians, and that the limited research funds are directed towards to these priorities.^2–4^ Priority-setting initiatives including patients and clinicians can influence the direction of future research and funding at the policy, institutional and research team levels.^5 6^ Examples of pioneer priority-setting partnerships were between Asthma UK and the British Thoracic Society.^7^ Addressing topics or relevance to patients and clinicians help reduce research waste, as highlighted by Chalmers *et al*.^8^

The aim of this study was to develop a list of priorities for glaucoma research involving clinicians and patients from different countries in Europe. This initiative was supported by the European Glaucoma Society (EGS).

## Materials and methods

**Figure 1:**
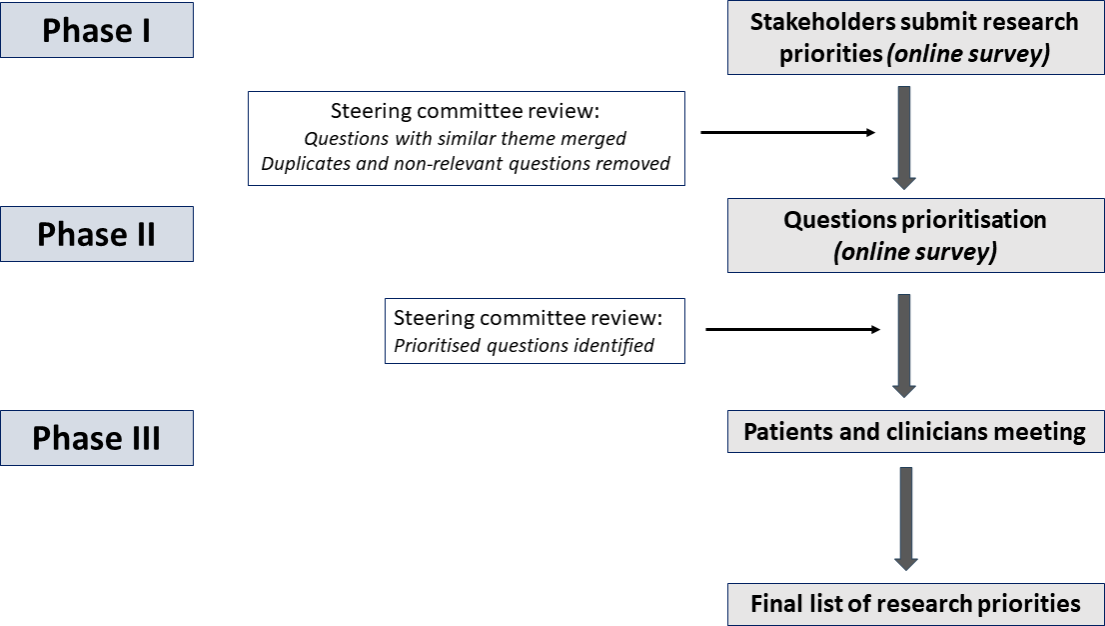
Flow chart describing phases of the study. Phases I and II were done in parallel among patients and glaucoma experts, independently.

A Steering Group was created among members of the EGS Scientific and Outcomes Committees. The purpose of the steering committee was to develop a protocol and facilitate work. We used a three-phase method, including a two-round electronic Delphi survey and a workshop (figure 1).^9 10^ The clinician and patient electronic surveys were conducted by email, in parallel and independently.

### Phase I: electronic survey to identify patient and clinician research priorities

Patient organisations in Europe were identified through a process of peer knowledge and consultation among the Steering Group members’ networks. Patients were also approached directly by their attending glaucoma specialist when attending a clinic to answer the survey. In phase I, patients were contacted by email or by supporting staff in the clinic waiting area and were asked a series of questions regarding their demographics, glaucoma treatment, perception of glaucoma care and research priorities. The survey was distributed to patients from 27 European countries from 3 to 24 May 2022. The survey consists of 23 questions divided into 3 sections. The survey was translated into six languages: English, French, German, Spanish, Portuguese and Greek.

Regarding the clinicians research priorities, the invited participants were EGS members, ophthalmologists with expertise in glaucoma care. The survey was sent to active and emeritus members (total n=788).

Submitted research questions were translated (if not in English language) and analysed by steering committee members (AT, PF). Text mining was performed to identify the most frequently used keywords from the translated patient responses. A frequency of word table and word cloud were generated. Similarly, themed responses were then merged by the two steering committee members to ensure the meaning of the priorities was not distorted. The steering committee members initially worked independently and then for cases of disagreement reached a consensus on categorisation of each priority. No limit was placed on the number of research priorities each patient could suggest and all were included in the analysis. A similar process was followed for merging similarly themed responses obtained from the EGS member survey. All analyses were performed using R Studio (V.12.0, RStudio, PBC, Boston, Massachusetts, USA).

### Phase II: electronic survey to rank patient and clinician research priorities

During phase II, EGS members and patients were invited, via email, to rank the questions identified in phase I using a Likert scale from 1 (lowest research priority) to 5 (highest research priority). Two reminders were sent via email over a 4-week period.

The steering committee reviewed the results. The mean rank score of the research priorities was calculated. Common and similar research priorities between clinicians and patients were merged but keeping the original description. The top 20 research priorities from both cohorts were selected. Similar questions were merged ensuring that the meaning of the questions was not distorted and keeping the original description to produce the top 20 joint research priorities carried forward to phase III.

### Phase III: meeting with patients and clinicians to reach consensus on top 10 priorities

The final priority setting stage (phase III) was a 1 day workshop in Lisbon on 30 September 2022 facilitated by an expert researcher (NMcC). The purpose of the workshop was to exchange knowledge and to make decisions about the most important research priorities, based on the wide set of experiences represented by the workshop participants, using an adapted Nominal Group Technique (NGT). NGT is appropriate when small groups want to make decisions within a limited period of time. The technique allows for consideration of everyone’s opinions through discussion and can incorporate both ranking and voting exercises. Participants were informed that the outcomes from the workshop would be shared with researchers and research funders. Eight patients and four clinicians from different European countries able to communicate well in English participated in the workshop. We tried to have a wide range of ages and gender balance among patients. In addition, there were two observers (current EGS President and EGS chair of European Union Committee) who did not participate in the discussions/rankings. The goal was to determine and rank the top 10 questions for research. All participants declared their interests. The role of the facilitator was to supervise group dynamics to ensure that all participant voices were heard and considered, to encourage debate and transparency and to help draw participants to consensus.

Before the workshop, participants were required to complete a ‘preworkshop exercise’, where they reviewed the 20 short-listed priorities identified by phase II. They were instructed to order these priorities from ‘1’ (most important area for research in your opinion) to ‘20’ (least important area for research in your opinion). At the workshop, following a short presentation and introduction, each participant was given the opportunity to share their ‘top 3’ and ‘bottom 3’ priorities with the group, and to explain the reasons for their rankings. These were noted by the facilitator. This completed the first part of the workshop. During a break, the workshop facilitator identified those priorities that were most often cited within the ‘top 3’ and ‘bottom 3’ by participants and arranged these in rough groups across a large table (using A4 cards printed with each priority, A–T). Other cited priorities, or those not mentioned by any participant, were arranged in a middle group. Participants then discussed the priorities and their order, until the top 10 priorities were ranked in order. On two occasions, a vote was taken to decide between the order of two priorities. The workshop discussions were recorded, with permission of participants.

## Results

### Phase I

**Figure 2:**
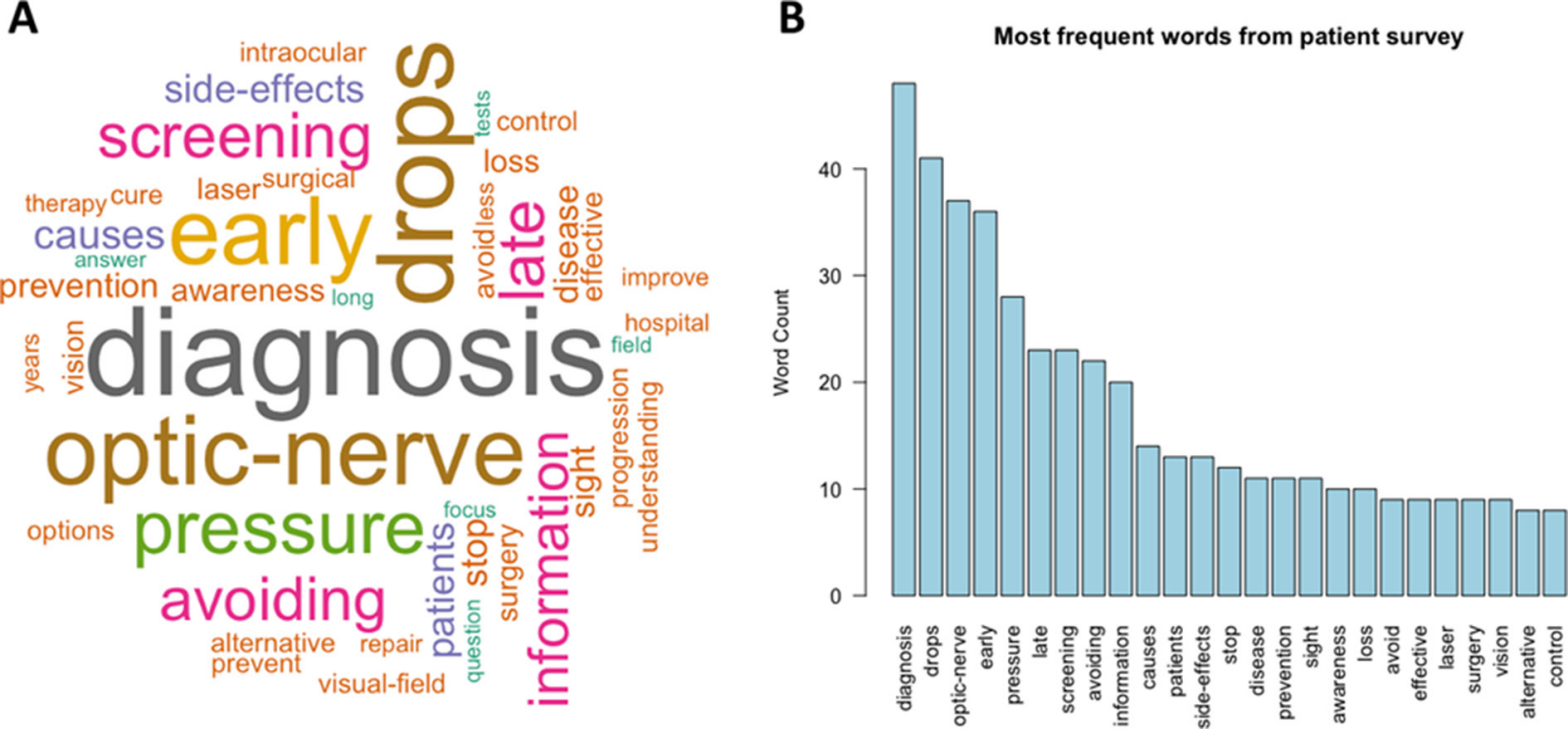
Word cloud (A) and bar chart (B) showing most frequent words used by patients responding to research priority question in phase I.

Of 402 patients from 20 European countries answering the questionnaire, 308 proposed one or more research priorities. One hundred fifty-one of 308 respondents (49.0%) were from the UK, 75 (24.4%) from France, 20 (6.5%) from Germany, with the remainder from other European countries. Of those proposing a research priority, 190 (61.7%) were female. Respondents’ age range was diverse, with 33 of 308 (10.7%) ≤49 years, 51 (16.7%) between 50 and 59 years, 87 (28.2%) between 60 and 69 years, 104 (33.8%) between 70 and 79 years and 33 (10.7%) 80 years or older. Two hundred twenty-eight (74.0%) were currently being treated with ocular hypotensive eye drops, 135 (43.8%) had undergone laser treatment for glaucoma and 132 (42.9%) had undergone glaucoma surgery. The most frequent words used by patients to describe the research priorities most important to them are summarised in figure 2.

**Figure 3:**
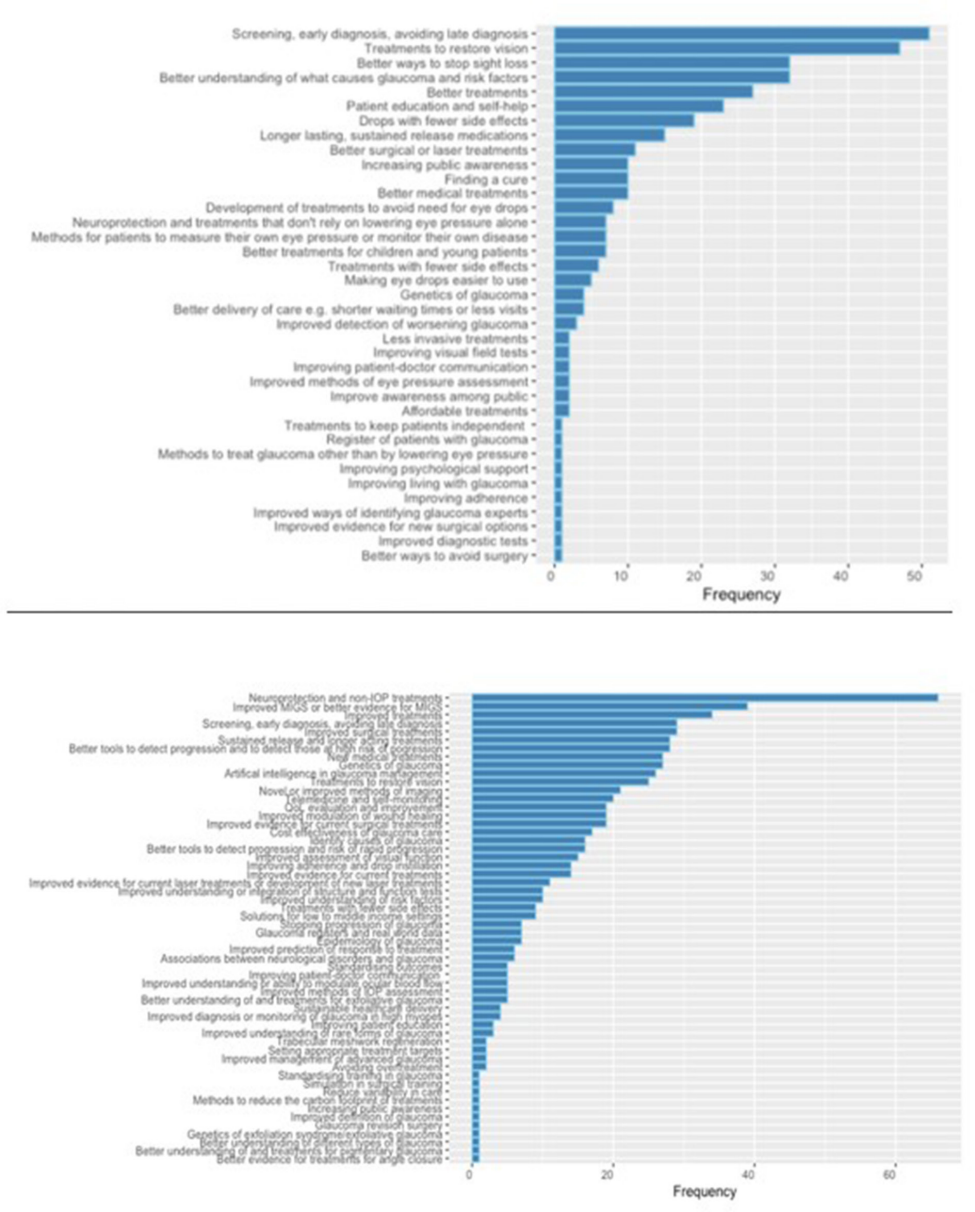
Top: frequency of research priorities proposed by patients responding to phase I. Bottom: frequency of research priorities proposed by clinicians responding to phase I.

The most commonly cited research priorities related to improving screening and early diagnosis (51 of 308, 16.6%), followed by treatments to restore vision (47 of 308, 15.3%), better ways to stop sight loss (32 of 308, 10.4%), improved understanding of risk factors for sight loss (32 of 308, 10.4%), better treatments (27 of 308, 8.8%), drops with fewer side effects (19 of 308, 6.2%) and improved resources for patient education and self-help (23 of 308, 7.5%) (figure 3).

**Figure 4:**
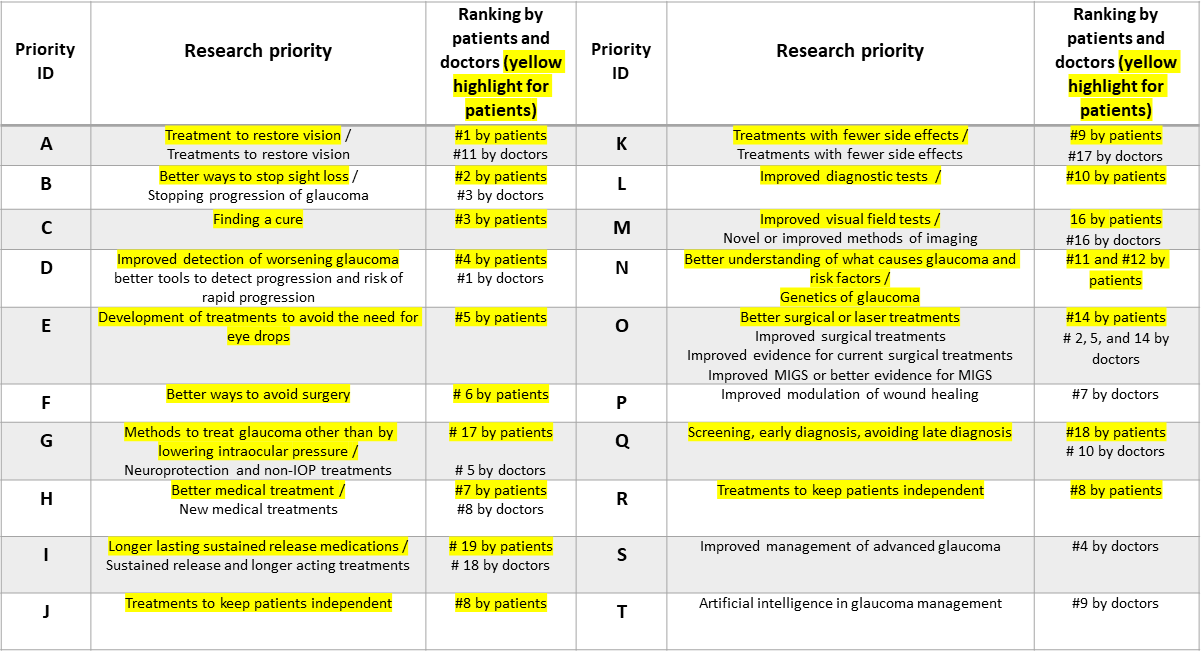
Top 20 research priorities identified in phase II according to clinicians and patients (highlighted in yellow). MIGS, minimally invasive glaucoma surgery.

A total of 150 clinicians proposed one or more research priorities. The priorities most commonly proposed by clinicians were neuroprotection (66 of 150, 44%), improved or better evidence for minimally invasive glaucoma surgery (39, 26%), improved treatments (34, 22.7%), screening, early diagnosis and avoiding late diagnosis (29, 19.3%), improved surgical treatments (29, 19.3%), better tools to detect progression and those at high risk of progression (28, 18.7%), sustained release and longer acting treatments (28, 18.7%), new medical treatments (27, 18%), artificial intelligence (26, 17.3%) and treatments to restore vision (25, 16.7%) (figure 4).

### Phase II

A total of 279 patients provided email contact details and were invited to participate in phase II. A total 111 of 279 responded (39.8% response rate), including 61 responding to the English language survey, 5 to the Spanish survey, 25 to the German survey and 20 to the French survey.

Patient and clinician round 2 scores are summarised in online supplemental appendix 1. The highest-ranking priority was treatments to restore vision (mean score 4.50), followed by better ways to stop sight loss (mean score 4.48), finding a cure (mean score 4.40), improved detection of worsening glaucoma (mean score 4.36), development of treatments to avoid need for eye drops (mean score 4.22) and better ways to avoid surgery (mean score 4.16).

10.1136/bjo-2023-323648.supp1 Supplementary data

A total of 147 clinicians provided their email details and were invited to participate in phase II; 65% (96 of 147) clinicians responded. Clinicians assigned the highest scores to research priorities; better tools to detect progression and risk of rapid progression (mean score 4.31), improved surgical treatments (mean score 4.18), stopping progression of glaucoma (mean score 4.12), improved management of advanced glaucoma (mean score 4.08), improved evidence for current surgical treatments (mean score 4.05) and neuroprotection (mean score 3.99) (online supplemental appendix 1).

The top 20 priorities scored by clinicians and patients are summarised in figure 4.

### Phase III

In phase III, patients (n=8) were from the following countries: the UK (n=3), France, Germany, Portugal, Sweden and Norway. There were five females and three males. Clinicians were from the UK (n=3) and Finland, with three males and one female.

Table 1 presents the agreed top 10 priorities for research that followed discussion during the workshop. Workshop attendees proposed that the following considerations should be taken into account when defining research priorities:

- The priority ‘finding a cure’ as an overall encompassing goal.
- The importance of ‘improving quality of life’ for people with glaucoma
- The priority ‘artificial intelligence in glaucoma management’ as a tool to achieve other priorities.
- Priority #1 (‘better ways to stop sight loss/stopping progression of glaucoma’) includes (but may not be limited to) priorities 4, 6 and 7.

**Table 1:** Top 10 research priorities identified in phase III

	Priority/Uncertainty
1.	Better ways to stop sight loss/stopping progression of glaucoma.
2.	Treatments to restore vision.
3.	Improved detection of worsening glaucoma/better tools to detect progression.
4.	New/Better medical treatments.
5.	Better understanding of what causes glaucoma and risk factors/genetics of glaucoma.
6.	Better surgical or laser treatments including improved MIGS or better evidence for MIGS.
7.	Methods to treat glaucoma other than lowering IOP/neuroprotection and non-IOP treatments.
8.	Improved diagnostic tests including 8(a) improved visual field tests/novel or improved methods of imaging.
9.	Screening, early diagnosis, avoiding late diagnosis.
10.	Treatments with fewer side effects.

MIGS, minimally invasive glaucoma surgery.

## Discussion

We have reported the results of a European-wide effort to establish the top 10 priorities for research in glaucoma. Our process has aimed at reflecting the priorities of patients and clinicians. Although we observed some overlap in topics, an important finding of our process is that patients and doctors have different priorities. For example, finding a cure was a top research priority by patients but not identified as such by doctors, possibly due to feasibility considerations. Patients’ priorities not shared by doctors included novel treatments to avoid the need for eye drops and to avoid surgery, and interventions to keep patients’ independence. Doctors’ priorities not considered important by patients included modulation of wound healing and the use of artificial intelligence.

In the final workshop, the two most important research priorities (treatments to stop sight loss and treatments to restore vision) were the ones identified by patients, reflecting the larger importance of patients’ voice. Some of the top 10 research priorities identified by clinicians (eg, use of artificial intelligence, improved modulation of wound healing) were not included in the final top 10 list after phase III discussions.

The strengths of this study are that it followed the robust standard methodology, and that we included a fairly large number of clinicians and patients from different European countries.^11^ Modified electronic Delphi process is commonly used to reach consensus and identify research priorities in diverse health areas.^12–16^

Several frameworks have been used to guide the process of priority setting, including the James Lind Alliance Priority Setting Partnership (JLA PSP),^17^ Essential National Health Research (ENHR)^18^ and the Dialogue Model.^19^ The JLA PSP method convenes patients, carers and clinicians to equally and jointly identify questions about healthcare that cannot be answered by existing evidence that are important to all groups (ie, research needs).^17^ The identified research needs are then prioritised by the groups resulting in a final list (often a top 10) of research priorities. Non-clinical researchers are excluded from voting on research needs or priorities but can be involved in other processes (eg, knowledge synthesis). The ENHR method, initially designed for health research priority setting at the national level, involves researchers, decision-makers, health service providers and communities throughout the entire process of identifying and prioritising research topics.^18 19^

This study has some limitations. First, the response rate among clinicians was low and thus may not be representative. It is possible a different design of the electronic survey or incentives may have improved the response rate. The patients who volunteered to participate in the survey may not be representative of the wider population of people with glaucoma. There may be an over-representation of patients with history of glaucoma surgery and presumably with severe stage of the disease, which may explain that the top research priority is ‘treatment to restore vision’. However, this was also among the top 20 priorities for clinicians which confirms the importance of this topic. The clinicians’ different preference is probably based on the understanding that the glaucoma damages are not reversible and research in this area will take a long time to be translated in improved outcomes. There was also a bias towards patients from the UK and France, with fewer patients included from other European countries. It is conceivable that differences in socioeconomic status, ethnicity, health beliefs, mode of healthcare provision and other factors could result in different priorities. Nevertheless, the research priorities identified in this study cover broad topics and to the best of our knowledge this was first attempt to identify research priorities in glaucoma across Europe.

In conclusion, the results of this study can be used to guide research funding bodies and the wider research community in advancing the quality of care for patients with glaucoma. An effort to identify specific research questions and define study designs (population, intervention, comparator, outcome) to address the identified research priorities is currently under way.

## Data Availability

Data sharing not applicable as no datasets generated and/or analysed for this study.
