# Supplementary material for: European Glaucoma Society research priorities for glaucoma care
Source: Br J Ophthalmol. 2023 Nov 3;108(8):1088–93. doi: 10.1136/bjo-2023-323648 (PMC11287634; doi:10.1136/bjo-2023-323648)
Supplement: Supplementary data [file bjo-2023-323648supp001.pdf]

EGS Research Priorities Delphi Survey - Round 2 Results Summary

Response rates

OPEN	Glaucoma research priorities for patients - French<br>Design Distribute Analyse	v1atatha@ed.ac.uk	20 of 68 (29%)	24 Aug 2022	1 Oct 2022	8 DAYS LEFT
OPEN	Glaucoma research priorities for patients - German<br>Design Distribute Analyse	v1atatha@ed.ac.uk	25 of 46 (54%)	24 Aug 2022	1 Oct 2022	8 DAYS LEFT
OPEN	Glaucoma research priorities for patients - Spain<br>Design Distribute Analyse	v1atatha@ed.ac.uk	5 of 18 (27%)	24 Aug 2022	1 Oct 2022	8 DAYS LEFT
OPEN	Glaucoma research priorities for patients<br>Design Distribute Analyse	v1atatha@ed.ac.uk	61 of 147 (41%)	24 Aug 2022	1 Oct 2022	8 DAYS LEFT
OPEN	EGS research priorities round 2<br>Design Distribute Analyse	v1atatha@ed.ac.uk	97 of 147 (65%)	24 Aug 2022	23 Sep 2022	

279 patients provided email and were invited to participate in round 2. 111 responded (39.8% response rate), including 61 responding to the English language survey, 5 to the Spanish survey, 25 to the German survey and 20 to the French survey.

147 clinicians provided their email and were invited to participate in round 2. 65% responded. 2 reminder emails were sent, only to those who did not respond to the initial email.

For round 2 patients were asked to score each research priority from 1 (least important) to 5 (most important).

Clinician Round 2 scores (0 to 5, with 5 being very important)

Priority	Mean
Better tools to detect progression and risk of rapid progression	4.309278
Improved surgical treatments	4.175258
Stopping progression of glaucoma	4.123711
Improved management of advanced glaucoma	4.082474
Improved evidence for current surgical treatments	4.051546
Neuroprotection and non IOP treatments	3.989691
Improved modulation of wound healing	3.979381
New medical treatments	3.969072
Artificial intelligence in glaucoma management	3.917526
Screening early diagnosis avoiding late diagnosis	3.886598
Improved evidence for current treatments	3.835052
Treatments to restore vision	3.835052
Glaucoma registers and real world data	3.814433
Improved MIGS or better evidence for MIGS	3.762887
Improved prediction of response to treatment	3.721649
Novel or improved methods of imaging	3.711340
Treatments with fewer side effects	3.670103
Sustained release and longer acting treatments	3.597938
Avoiding overtreatment	3.587629
Quality of life evaluation and improvement	3.587629
Setting appropriate treatment targets	3.567010
Standardising outcomes	3.556701
Glaucoma revision surgery	3.536082
Increasing public awareness	3.525773
Improved understanding of risk factors	3.515464
Identify causes of glaucoma	3.505155
Better evidence for treatments for angle closure	3.474227
Improved understanding or integration of structure and function tests	3.463918
Trabecular meshwork regeneration	3.422680
Improved evidence for current laser treatments or development of new laser treatments	3.412371
Standardising training in glaucoma	3.391753
Genetics of glaucoma	3.371134
Solutions for low to middle income settings	3.371134
Improved assessment of visual function	3.360825
Telemedicine and self monitoring	3.360825
Improving patient education	3.340206
Better understanding of and treatments for secondary glaucomas	3.309278
Sustainable healthcare delivery	3.309278
Cost effectiveness of glaucoma care	3.257732
Improved methods of IOP assessment	3.257732
Improving adherence and drop instillation	3.257732
Improved understanding or ability to modulate ocular blood flow	3.175258
Reduce variability in care	3.164948
Improving patient doctor communication	3.082474
Improved understanding of rare forms of glaucoma	2.845361
Improved definition of glaucoma	2.824742
Methods to reduce the carbon footprint of treatments	2.742268

Patient responses (pooled from all countries)

Priority	Mean
Treatments to restore vision	4.495495
Better ways to stop sight loss	4.477477
Finding a cure	4.400000
Improved detection of worsening glaucoma	4.360360
Development of treatments to avoid need for eye drops	4.216216
Better ways to avoid surgery	4.162162
Better medical treatments	4.135135
Treatments to keep patients independent	4.135135
Treatments with fewer side effects	4.135135
Improved diagnostic tests	4.099099
Better understanding of what causes glaucoma and risk factors	4.064220
Genetics of glaucoma	4.036036
Better treatments for children and young patients	4.018018
Better surgical or laser treatments	3.990909
Better delivery of care e g shorter waiting times or less visits	3.954955
Improving visual field tests	3.945946
Methods to treat glaucoma other than by lowering eye pressure	3.918919
Screening early diagnosis avoiding late diagnosis	3.900000
Longer lasting sustained release medications	3.882883
Improved evidence for new surgical options	3.855856
Methods for patients to measure their own eye pressure or monitor their own disease	3.792793
Improving living with glaucoma	3.720721
Improving patient doctor communication	3.702703
Less invasive treatments	3.702703
Drops with fewer side effects	3.693694
Improved ways of identifying glaucoma experts	3.666667
Patient education and self help	3.657658
Improved methods of measuring eye pressure	3.630631
Affordable treatments	3.612613
Improve awareness among public	3.536364
Increasing public awareness	3.486486
Making eye drops easier to use	3.387387
Improving psychological support	3.369369
Register of patients with glaucoma	3.243243
Improving adherence	3.203704

Patient responses – Round 1

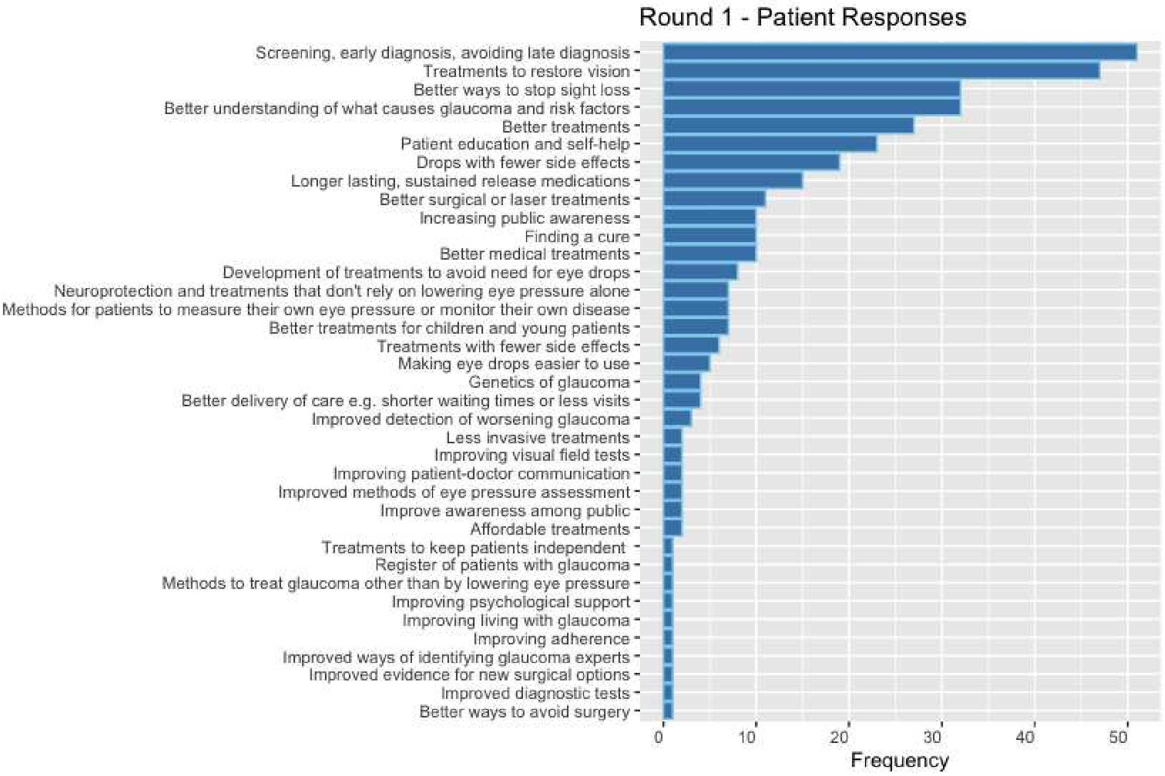

Patient responses – Round 1

	Priority	Frequency
1	Affordable treatments	2
2	Better delivery of care e.g. shorter waiting times or less visits	4
3	Better medical treatments	10
4	Better surgical or laser treatments	11
5	Better treatments	27
6	Better treatments for children and young patients	7
7	Better understanding of what causes glaucoma and risk factors	32
8	Better ways to avoid surgery	1
9	Better ways to stop sight loss	32
10	Development of treatments to avoid need for eye drops	8
11	Drops with fewer side effects	19
12	Finding a cure	10
13	Genetics of glaucoma	4
15	Improve awareness among public	2
16	Improved detection of worsening glaucoma	3
17	Improved diagnostic tests	1

18	Improved evidence for new surgical options	1
19	Improved methods of eye pressure assessment	2
20	Improved ways of identifying glaucoma experts	1
21	Improving adherence	1
22	Improving living with glaucoma	1
23	Improving patient-doctor communication	2
24	Improving psychological support	1
25	Improving visual field tests	2
26	Increasing public awareness	10
27	Less invasive treatments	2
28	Longer lasting, sustained release medications	15
29	Making eye drops easier to use	5
30	Methods for patients to measure their own eye pressure or monitor their own disease	7
31	Methods to treat glaucoma other than by lowering eye pressure	1
32	Neuroprotection and treatments that don't rely on lowering eye pressure alone	7
33	Patient education and self-help	23
34	Register of patients with glaucoma	1
35	Screening, early diagnosis, avoiding late diagnosis	51
38	Treatments to keep patients independent	1
39	Treatments to restore vision	47
40	Treatments with fewer side effects	6

Summary of Round 1 – Clinician Responses

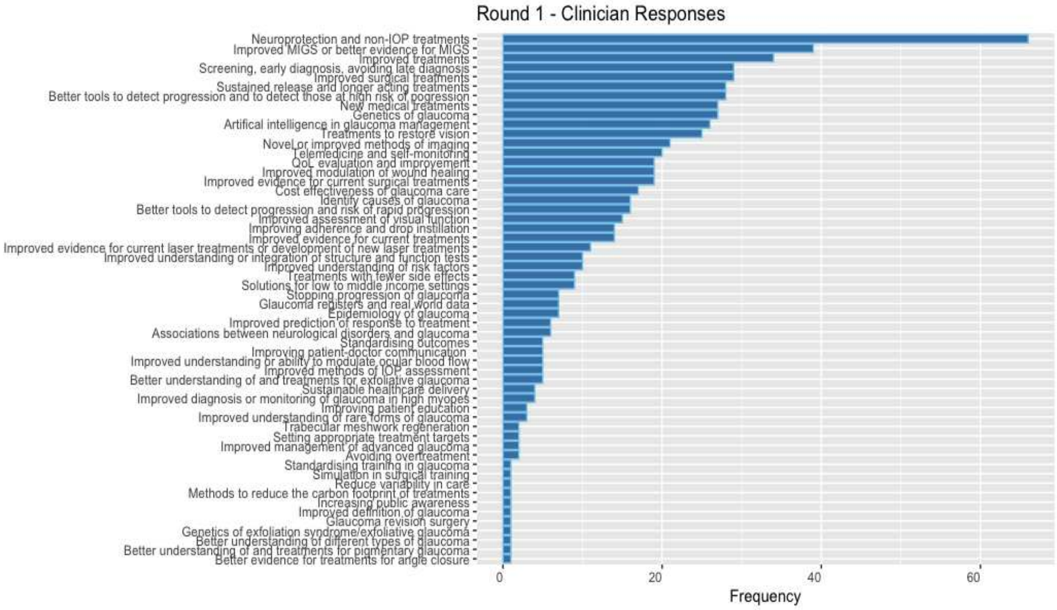

Summary of Round 1 – Clinician Responses

Priority	Frequency
Artifical intelligence in glaucoma management	26
Associations between neurological disorders and glaucoma	6
Avoiding overtreatment	2
Better evidence for treatments for angle closure	1
Better tools to detect progression and risk of rapid progression	16
Better tools to detect progression and to detect those at high risk of pogression	28
Better understanding of and treatments for exfoliative glaucoma	5
Better understanding of and treatments for pigmentary glaucoma	1
Better understanding of different types of glaucoma	1
Cost effectiveness of glaucoma care	17
Epidemiology of glaucoma	7
Genetics of exfoliation syndrome/exfoliative glaucoma	1
Genetics of glaucoma	27
Glaucoma registers and real world data	7
Glaucoma revision surgery	1
Identify causes of glaucoma	16
Improved assessment of visual function	15
Improved definition of glaucoma	1

Improved diagnosis or monitoring of glaucoma in high myopes	4
Improved evidence for current laser treatments or development of new laser treatments	11
Improved evidence for current surgical treatments	19
Improved evidence for current treatments	14
Improved management of advanced glaucoma	2
Improved methods of IOP assessment	5
Improved MIGS or better evidence for MIGS	39
Improved modulation of wound healing	19
Improved prediction of response to treatment	6
Improved surgical treatments	29
Improved treatments	34
Improved understanding of rare forms of glaucoma	3
Improved understanding of risk factors	10
Improved understanding or ability to modulate ocular blood flow	5
Improved understanding or integration of structure and function tests	10
Improving adherence and drop instillation	14
Improving patient education	3
Improving patient-doctor communication	5
Increasing public awareness	1
Methods to reduce the carbon footprint of treatments	1
Neuroprotection and non-IOP treatments	66
New medical treatments	27
Novel or improved methods of imaging	21
QoL evaluation and improvement	19
Reduce variability in care	1
Screening, early diagnosis, avoiding late diagnosis	29
Setting appropriate treatment targets	2
Simulation in surgical training	1
Solutions for low to middle income settings	9
Standardising outcomes	5
Standardising training in glaucoma	1
Stopping progression of glaucoma	7
Sustainable healthcare delivery	4
Sustained release and longer acting treatments	28
Telemedicine and self-monitoring	20
Trabecular meshwork regeneration	2
Treatments to restore vision	25
Treatments with fewer side effects	9
